# Ferritin Nanocages Exhibit Unique Structural Dynamics When Displaying Surface Protein

**DOI:** 10.3390/ijms26157047

**Published:** 2025-07-22

**Authors:** Monikaben Padariya, Natalia Marek-Trzonkowska, Umesh Kalathiya

**Affiliations:** International Centre for Cancer Vaccine Science, University of Gdansk, ul. Kładki 24, 80-822 Gdansk, Poland; natalia.marek-trzonkowska@ug.edu.pl

**Keywords:** nanocage, ferritin, vaccine, SARS-CoV-2, host receptor, surface protein, dynamics

## Abstract

Ferritin nanocages with spherical shells carry proteins or antigens that enable their use as highly efficient nanoreactors and nanocarriers. Mimicking the surface Spike (S) receptor-binding domain (RBD) from SARS-CoV-2, ferritin nanocages induce neutralizing antibody production or block viral entry. Herein, by implementing molecular dynamics simulation, we evaluate the efficiency in the interaction pattern (active or alternative sites) of H-ferritin displaying the 24 S RBDs with host-cell-receptor or monoclonal antibodies (mAbs; B38 or VVH-72). Our constructed nanocage targeted the receptor- or antibody-binding interfaces, suggesting that mAbs demonstrate an enhanced binding affinity with the RBD, with key interactions originating from its variable heavy chain. The S RBD interactions with ACE2 and B38 involved the same binding site but led to divergent dynamic responses. In particular, both B38 chains showed that asymmetric fluctuations had a major effect on their engagement with the Spike RBD. Although the receptor increased the binding affinity of VVH-72 for the RBD, the mAb structural orientation on the nanocage remained identical to its conformation when bound to the host receptor. Overall, our findings characterize the essential pharmacophore formed by Spike RBD residues over nanocage molecules, which mediates high-affinity interactions with either binding partner. Importantly, the ferritin-displayed RBD maintained native receptor and antibody binding profiles, positioning it as a promising scaffold for pre-fusion stabilization and protective RBD vaccine design.

## 1. Introduction

The global coronavirus disease 2019 (COVID-19) pandemic caused by the outbreak of severe acute respiratory syndrome coronavirus 2 (SARS-CoV-2) affected more than 700 million people, with over 6 million deaths [1,2,3]. SARS-CoV-2 is a single- and positive-stranded RNA virus with a large RNA genome that encodes a non-structural replicase polyprotein and four structural proteins: Spike (S), envelope (E), membrane (M), and nucleocapsid (N) proteins [4,5,6]. Among these structural proteins, the glycosylated Spike (S protein) is particularly important as the main antigenic component responsible for inducing host immune responses and neutralizing antibodies; thus, it is a prominent target for antiviral vaccine development [7,8,9]. The S protein plays a pivotal role in viral infection and pathogenesis by recognizing and binding to host-cell receptors, such as Angiotensin-Converting Enzyme 2 (ACE2), which mediates SARS-CoV-2 entry into host cells [10]. Glycosylation is the most common post-translational modification (PTM) in viruses [11], and it facilitates viral protein folding, intracellular trafficking, and modulation of interactions with host receptors. Additionally, glycosylation influences innate and adaptive immune responses by altering host recognition, viral replication, and infectivity.

During viral infection, the S protein is cleaved by host-cell proteases into S1 and S2 subunits. The receptor-binding domain (RBD) within S1 interacts with ACE2, making the S protein essential for SARS-CoV-2 infectivity and transmissibility [12,13]. A variety of virus-based, subunit-based, and RNA- or DNA-based vaccines have been used to develop a safe and effective SARS-CoV-2 vaccine [14]. Avoiding the premature degradation of molecules and adjuvant booster-shot requirements are among the advantages of RNA- or DNA-based vaccines [15,16]. To overcome the limitations associated with these vaccines, nanocarrier-based vaccine delivery was considered, involving inorganic, lipid, polymeric, virus-like, micelle, and protein nanoparticles that improve the antigen structure and stability [16,17]. Protein nanoparticles are considered potential candidates for vaccine nanobiotechnology, owing to their biocompatibility and flexibility of design thanks to protein engineering [18]. One such naturally occurring protein nanoparticle, which is highly appealing as a SARS-CoV-2 vaccine carrier, is ferritin, a ubiquitous iron storage and detoxification protein that protects cells from iron-induced oxidative damage [19]. The ferritin protein nanocage, with its inherent self-assembly properties, demonstrates exceptional chemical and thermal stability, a reversible assembly–disassembly dynamic, and versatile surface functionalization via chemical or genetic methods. These attributes make ferritin as ideal scaffold for antigen presentation in vaccine development [17,19].

A wide range of alternative drug nanocarriers exists, including liposomes’ polymeric and albumin-bound nanoparticles, compared to which ferritin is relatively new; however, it is attracting research attention and is reported to hold potential [20,21]. Generally, nanocarriers have been made of natural and artificial materials, such as inorganic materials including Au and Fe_3_O_4_, and organic materials including proteins, lipids, and PLGA [21]. Ferritin is safer compared to most of the other nanocarriers due to its mammalian or human source and protein nature. It has low immunogenicity and is found to be highly biocompatible [21,22]. Metal-containing nanocarriers as well as charged liposomes are found to be toxic; their non-biodegradability and undesirable biocompatibility cause chronic inflammation [21,23,24]. To enhance the immunogenicity of recombinant protein/peptide-based vaccines, an effective strategy is to employ a potential antigen delivery system. Self-assembled ferritin is well-suited to displaying antigens of interest at a high density on its surface by gene fusion, thereby enhancing antigen immunogenicity. Ferritin nanoparticle platforms have been widely incorporated to display antigens from various viruses, including influenza viruses, respiratory syncytial virus, HIV, and SARS-CoV-2, and they elicit more potent and effective immune responses than soluble antigens [25]. Strong immune responses observed in pre-clinical studies against various pathogens have led to the development of ferritin nanoparticle-based vaccines in multiple phase I clinical trials [17].

The ferritin platform has potential in vaccine development as a carrier for drug delivery with its multimeric nature and ease of genetic modification, and it has also been reported to have barely any toxic side effects. A number of assessment models of protein particle-based vaccines have been employed on the market, mostly utilizing virus-like particles [26]. For instance, an in vivo study by Charlton et al. [27,28] evaluated the toxicity of horse spleen ferritin and showed that ferritin nanoparticles administered at doses intended for targeted MRI molecular imaging caused no short-term mortality or morbidity and had no significant effect on the structure or function of vital organs in mice. In vitro studies [29,30] utilized MTT viability assays on an infectious hematopoietic necrosis virus ferritin-based vaccine, and ferritin nanoparticles in zebrafish liver cells had no cytotoxicity up to 100 µg/mL. This represents the most important biomedical application of nanoparticles with a high degree of compatibility with living cells, which makes them a reasonable choice for mammalian and human vaccines [28]. Additionally, Han et al. [26,30] incorporated ferritin as an antigen (derived from ovalbumin) delivery nanoplatform for dendritic cell-based vaccine development, which induced antigen-specific immune responses.

A distinct class of ferritin nanocages was proposed to offer a versatile and potent platform for the coronavirus vaccines, which can enhance the display of multivalent antigens against different SARS-CoV-2 variants. In animal models, ferritin nanoparticles displaying the S RBD were found to enhance neutralizing antibodies, production (inducing the CD4 response), which can result in protection against coronavirus infection [31]. Alongside these, mosaic ferritin-based vaccines displaying Spike protein over their surface were found to be active against SARS-CoV-2, SARS-CoV, and MERS-CoV infection [32]. Moreover, cryo-EM studies have demonstrated the significance of the folding and antigenicity of these nanocages with Spike over their surface, which enhances neutralizing antibodies. These single-dose vaccine candidates can be lyophilized, maintaining their immunogenicity [33]. Alongside directly representing a surface protein, ferritin was found to evolve, displaying peptides or antigens towards MHC class I and II (major histocompatibility complex) molecules, which induce T-cell responses [34]. Various ferritins, including H-ferritin, L-ferritin, *Helicobacter pylori* ferritin (Hp-ferritin), and *Archaeoglobus fulgidus* ferritin, have recently been utilized for different applications. Among these, the two structurally and functionally closest relatives, Hp-ferritin and H-ferritin, are primarily used for antigen display. While Hp-ferritin facilitates trimeric antigen presentation, H-ferritin enables a monomeric display of antigen or protein. For most biomedical applications, H-ferritin is the preferred platform due to its high stability, excellent yield, and ease of engineering.

Molecular understanding and potential usages of ferritin-based vaccines were put forward in previous studies, which proposed that they may effectively inhibit viral entry by blocking the Spike-ACE2 network, and may induce cross-protective antibody responses [35,36,37]. Our study reports, for the first time, the structural dynamics and binding mechanism of the SARS-CoV-2 Spike RBD with the ACE2 host-cell receptor and antibodies when displayed over H-ferritin nanocages. The Spike RBD that directly binds with the ACE2 receptor is shown to present a potential therapeutic target against SARS-CoV2 infection. In addition, it is known that antibodies targeting the Spike RBD can neutralize infection by blocking viral entry. To assess such a mechanism, we evaluated the binding interactions of distinct antibodies, including B38 (docks at the RBD-ACE2 interface) and VVH-72 (or VVH; binds RBD while leaving the ACE2 site accessible), with RBD-displaying ferritin nanocages. Our findings demonstrate that ferritin nanocages representing the S RBD over their surface enable different states of conformations, which can enhance its binding with receptor or neutralizing antibodies, making it an attractive platform for pre-fusion (variant proof) RBD-based vaccine development. In addition, we report the potential usage of such a ferritin-based technique to obtain potential vaccine candidates against SARS-CoV-2 infection.

## 2. Results and Discussion

The versatile 24-subunit H-ferritin nanocage offers superior biocompatibility [15,38,39] and self-assembles into a spherical nanocage (~12 nm diameter) that enables multivalent antigen presentation and elicits a robust immune response. We simulated four distinct ferritin-based systems displaying Spike RBD binding with different monoclonal antibodies (mAbs: B38 and VVH-72), in the presence or absence of the ACE2 receptor. In our study, we used the optimized H-ferritin nanocages retrieved after 100 ns molecular dynamics simulations (MDSs) displaying the SARS-CoV-2 Spike RBD (T333-P521) linked with a stable GGGGS linker [35,37]. The active ‘up’ conformation of the S RBD (pdb id.: 6lzg [36,37]) was used for docking with ACE2 and antibodies (B38, pdb id.: 7bz5 [40]; VHH-72, pdb id,: 6waq [41]). Different modeled complexes were validated by superposition with the crystallographic structure, ensuring model prediction fidelity. While variability in the sequence can be observed for the Spike RBD [35] when with B38 (pdb id.: 7bz5 [40]) or VHH-72 (pdb. id.: 6waq [41]), our objective was to evaluate the binding pattern of the original Wuhan-Hu-1 strain of SARS-CoV-2 with ACE2 and mAbs when displayed over a nanocage. Our findings lead us to propose the mimicking model of viral attachment to the host-cell receptor, by displaying the RBDs over nanocages that can block ACE2 binding with SARS-CoV-2 virus and induce the production of neutralizing antibodies.

### 2.1. Nanocages Displaying S RBD Sharing a Common Interface with Its Target

**Figure 1:**
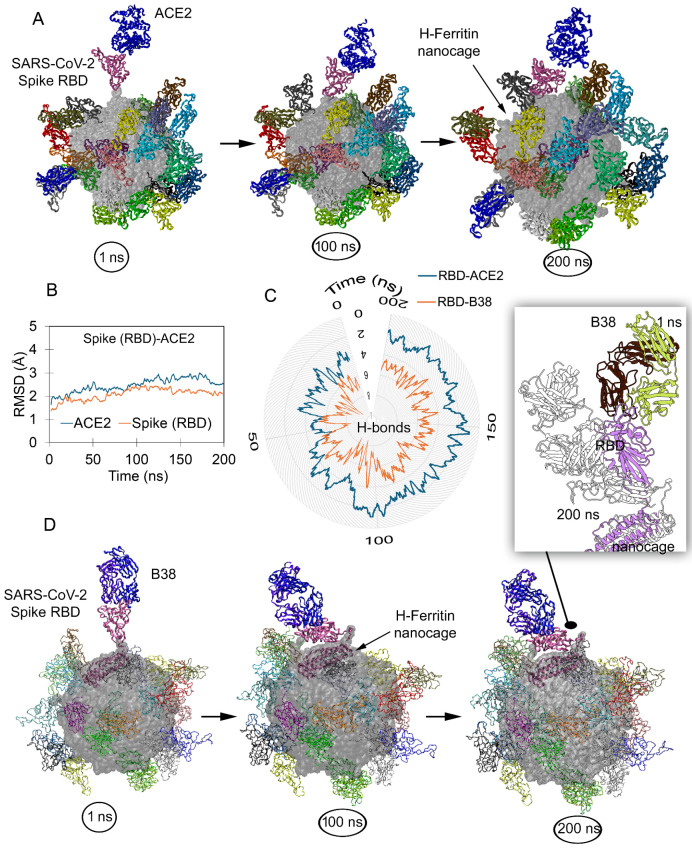
SARS-CoV-2 Spike receptor binding domain (RBD) over the H-ferritin nanocage. (**A**) The 24 ferritin monomers presenting the RBD over its surface, including a Spike monomer docked with the ACE2 receptor (pdb id.: 6lzg [36,37]). Individual conformation states gained by the Spike RBD-ACE2 during the 200 ns of molecular dynamics simulation (MDS) time are highlighted. (**B**) The measured stability of ACE2 (chain Y; labeled as per our MD systems) and Spike RBD (chain N) based on RMSD (Root Mean Square Deviation), computed on all atoms excluding hydrogens. In particular, for the Spike RBD, the RMSDs were computed for the T333-P521 residue range. (**C**) The time dependence interactions between Spike RBD and B38 (monoclonal antibody; mAb) or ACE2 over the nanocage. (**D**) The dynamics of the B38-Spike RBD system over the ferritin (pdb id.: 7bz5 [40]) nanocage, and the top right panel presents the structural convergence of the Spike RBD-ACE2 complex from 1 ns (RBD in violet, B38 heavy chain in brown, and light chain in green color) to 200 ns (displayed in silver) of MD simulations. ACE2 or B38 is shown in blue, and the 24 S RBDs are in different colors; in particular, RBD interacting with a receptor or antibody is displayed in mauve, and the H-ferritin nanocages are presented as surface in gray.

Previous studies have demonstrated that the SARS-CoV-2 Spike RBD maintains its ‘up’ active conformation when displayed on an H-ferritin nanocage [34,35,36,37]. The conformational states obtained from our previous study served as an initial configuration to investigate the binding efficiency with both ACE2 and monoclonal antibodies. Visualization of the 200 ns MD simulation trajectory (Video S1) demonstrated that the RBDs of our ferritin-based construct maintained their native binding orientation with ACE2, preserving key interaction sites (Figure 1A). The stability measured (based on RMSD; Root Mean Square Deviation) for protein–protein interacting (PPI) partners over the ferritin nanocage revealed that the Spike RBD and ACE2 fluctuate in the relaxed conformation ranging around 2 Å, which shows the protein reaching a stable state. While the S RBD was interacting with the ACE2 receptor in our simulated system, the other twenty three S RBD monomers over the ferritin nanocage maintained their ‘up’ active state with a possibility to dock a receptor or antibody. An analysis of residue interactions between nanocages displaying the S RBD and ACE2 revealed the retention of approximately three hydrogen bonds (H-bonds) during stable binding phases, despite an initial count nearly twice as high (Figure 1B,C). Such a binding pattern mirrors the observations from previous simulations of isolated RBD-ACE2 complexes [42], indicating that while the nanocage presentation maintains fundamental interaction dynamics, it provides stabilization through the multivalent display platform.

**Figure 2:**
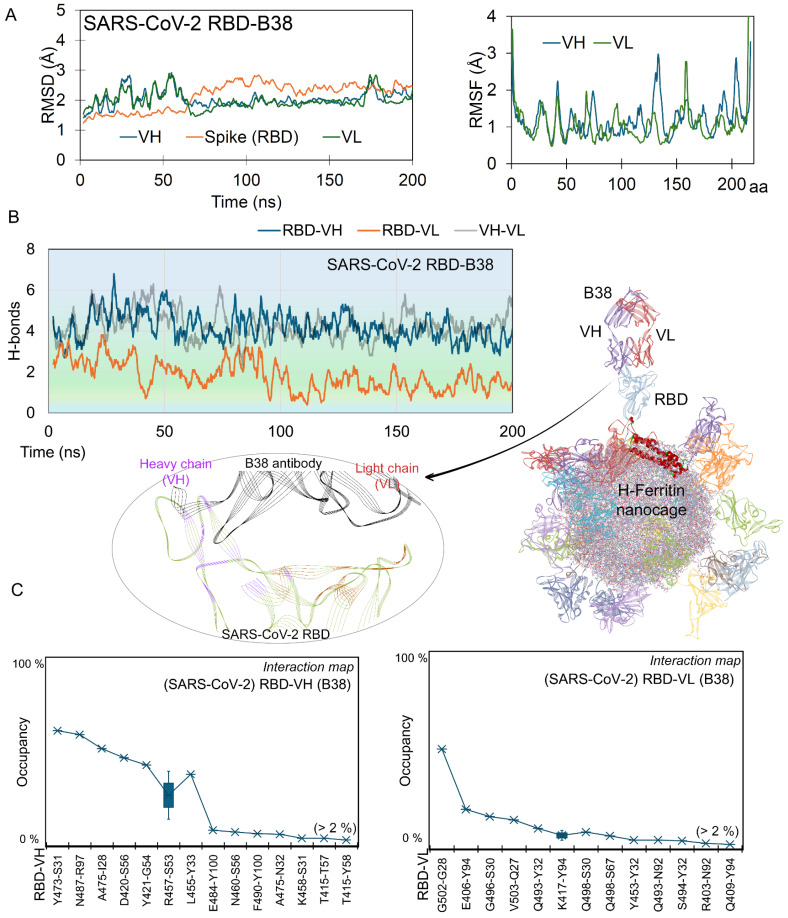
Distributed interaction pattern of B38 antibody (heavy chain; VH, and light chain; VL) with the Spike RBD over the H-ferritin nanocage. (**A**) The RMSDs of Spike RBD (chain N; residues 333–521) and B38 variable heavy (VH; chain Y) and variable light chains (VL; chain Z), and the right panel represents the Root Mean Square Fluctuation (RMSF; based on Cα atoms) of both chains from the monoclonal antibody. (**B**) The hydrogen bond (H-bond) interactions between SARS-CoV-2 RBD and B38 (VH or VL) antibody, which targets the RBD interface responsible for docking the ACE2 receptor. In addition, the interaction between VH and VL from B38 over 200 ns of MD simulation time was examined. The bottom panel represents the interacting regions emerging from the Spike RBD with B38 antibody. The S RBD is displayed in green, and its interactions with B38 VH are in purple and VL in red, while B38 is displayed in black. Occupancy defines a hydrogen bond interaction lasting more than 2% of the 200 ns MD simulation, and it was computed using a donor–acceptor distance cutoff of 3.5 Å and donor–hydrogen–acceptor angle cutoff of 160–180°. The B38 VL chain is shown in red and the VH chain in purple, and the 24 S RBDs are in different colors; in particular, RBD interacting with an antibody is in a light blue color, and the H-ferritin nanocage is displayed as lines. (**C**) High-occupancy (over 200 ns) interactions between SARS-CoV-2 Spike RBD with heavy and light chains of B38 antibody. Residue pairs occurring with different occupancy ranges of interactions are marked with a range.

The B38 antibody (pdb id.: 7bz5 [40]) competitively binds to the S RBD at the ACE2 interface, thereby blocking viral attachment to the host cell. Given such a neutralizing mechanism, we performed comparative analyses of both protein–protein (RBD-ACE2) and protein–antibody (RBD-B38) interactions (Figure 1D). Similar to ACE2 over the RBD-ferritin system, the B38 (VH; variable heavy chain or VL; variable light chain) antibody maintained its interactions with the S RBD (Figure 1D); however, a slight change from the initial position for B38 was observed (Video S2 and Figure 1D). Considering the binding behavior of the monoclonal antibody, we assessed the stability of the S RBD interacting partners such as the ACE2 receptor and VH or VL chains of B38 by measuring RMSD and RMSF (Root Mean Square Fluctuation; Figure 2A). The Spike RBD remained highly stable throughout the MD simulation, when complexed with the B38 antibody. In contrast, the VH and VL chains of B38 exhibited initial jumps in RMSD values, which were stabilized by the end of the simulation at ~2 Å, indicating conformational adjustments upon binding. Residue-level stability analysis (RMSF) revealed an asymmetric fluctuation between two B38 chains, suggesting differential flexibility in their interaction with the RBD (Figure 2A). Spike RBD monomers over ferritin were found to maintain a higher number of interactions with neutralizing antibodies, compared to its mapping with the ACE2 receptor (Figure 1D and Figure 2). The constructs of S RBD-B38 displayed over nanocages formed an average of ~6 hydrogen bonds throughout the simulation. When decomposing these interactions emerging from the B38 light or heavy chains with RBD, it was observed that VH generated a higher number of interactions compared to VL (Figure 2B). Both VH and VL chains from B38 mAb were found heavily interacting with each other over the nanocage, when docked with the Spike RBD. In addition, the regions emerging from the Spike RBD making contact with B38 antibodies were identified and are shown in Figure 2B.

High-frequency networking residues (>2% over 200 ns) between B38 and the Spike RBD over ferritin were monitored, which suggested that VH formed higher numbers of residue pairs with the RBD compared to VL (Figure 2C and Video S2). In particular, the Y473, N487, A475, D420, Y421, R457, L455, and R457 Spike RBD residues evolved, forming high-occupancy interactions with the heavy chain (VH) of B38, and G502, E406, G496, V503, and Q493 were found docking with the light chain (VL; Figure 2C). These high-frequency residues from the RBD over nanocages mimicking the B38 or ACE2 interactions were found to correlate with the crystal structure (pdb id.: 7bz5 [40]) and mutation landscape studies [35,37]. These residues of the Spike RBD forming interactions with B38 were evaluated with respect to distinct SARS-CoV-2 variants (Alpha, B.1.1.7; Beta, B.1.351; Delta, B.1.617.2 and B.1.617; Gamma, P.1; Lambda, C.37; and Omicron, B.1.1.529). In our previous study, we evaluated site-specific mutations at positions K417N/T, S477N, T478K, E484Q/A/K, G496S, Q498R, N501Y, and Y505H aa (amino acids) in the Spike RBD, which can influence its binding affinity with the B38 antibody over the H-ferritin nanocage [35]. A comparison of the MD-simulated structure with the best-docked model from our previous work [35] revealed Spike RBD residues Q493 and G496 in both, forming interactions with antibody B38. Such differences between simulated and docking model structures can be correlated with the motion throughout MD simulation, to distinguish the detailed interaction pattern over time. Among these systems, the L455, Y473, A475, N487, Q493, G496, and G502 residues from Spike RBD were found interacting with the B38 antibody, defining the hot-spot regions.

### 2.2. Simultaneous Approach of Antibody and Receptor Towards the Spike over the Nanocage

**Figure 3:**
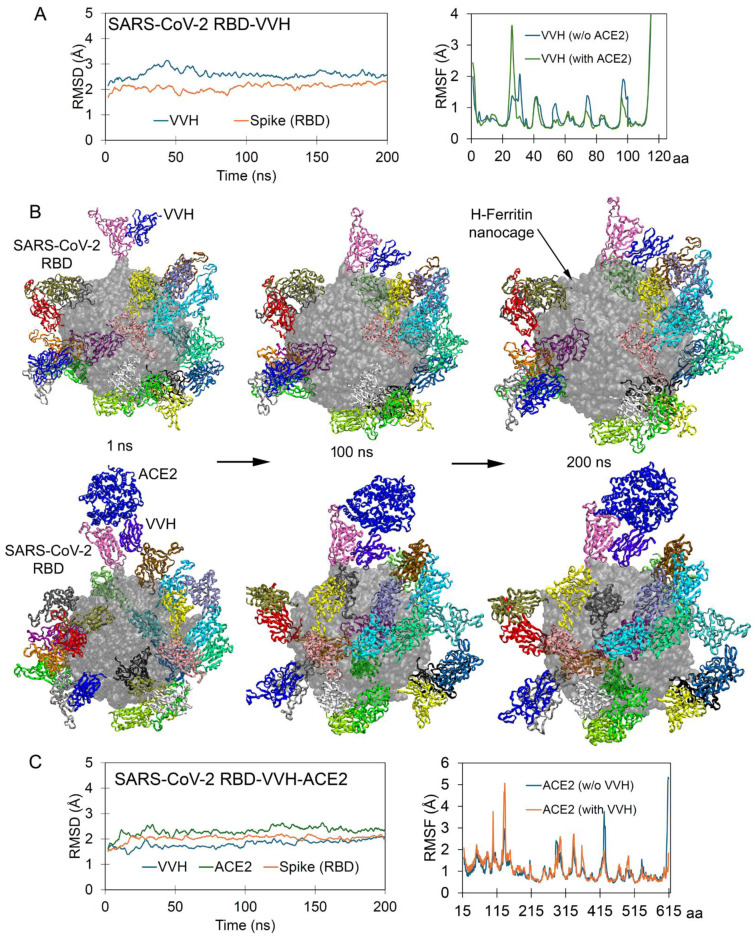
Simultaneous binding of VVH-72 antibody and ACE2 with Spike RBD. (**A**) The measured stability (RMSD) of the Spike RBD-VVH-72 system. The right panel represents the RMSF of an individual residue of mAb (VVH-72) in the presence or absence of ACE2. (**B**) Conformational change of VVH-72 antibody (pdb id.: 6waq [41]) and ACE2 with Spike RBD over the H-ferritin nanocage. The VVH-72 antibody simulated with RBD in the presence or absence of the ACE2 receptor, individual protein, and mAb coordinates were extracted over different time intervals. VVH-72 or ACE2 is shown in blue, and the 24 S RBDs are in different colors; in particular, the S RBD interacting with a receptor or antibody is in mauve, and the H-ferritin nanocage is displayed as the surface in gray. (**C**) The stability of the SARS-CoV-2 RBD(chain N)-VVH-ACE2 system, and the right panel demonstrates ACE2 (chain Y) residue fluctuation in the presence or absence of the VVH-72 (chain Z) antibody.

The conventional B38 antibody (~150 kDa) binds directly to surface of the Spike RBD, whereas several antibodies do not interfere with Spike-ACE2 interaction but bind to different regions of the RBD, such as the VVH-72 monoclonal antibodies (also termed nanobodies) [41]. It has been reported that the VVH-72 antibody and ACE2 can form simultaneous interactions with the Spike RBD. To investigate the interaction pattern of our designed nanocage constructs presenting surface protein, we evaluated the change in the binding affinity between partners within the antibody-ACE2-RBD-ferritin system (Figure 3). The RMSD values for both the RBD and VHH-72 antibody (system without ACE2) remained stable at approximately 2 Å during MD simulation. The presence of ACE2 stabilized most antibody residues, except for residues 20–35, which exhibited increased flexibility. This suggests potential allosteric regulation of the monoclonal antibody upon ACE2 binding (Figure 3A). Visualizing the dynamics of the VVH-72-RBD-ferritin (in the absence of ACE2; Video S3) system revealed that the presence of a monoclonal antibody led to the persistence of the ‘up’ active conformation of the RBD that can dock with receptor. In particular, the VVH-72 antibody was found to form a similar conformation for interacting with the Spike RBD over the nanocage in the presence or absence of the ACE2 receptor (Figure 3B). Notably, in the RBD-ACE2-VHH system displayed over the H-ferritin nanocage, the VHH-72 antibody exhibited greater stability compared to when complexed without ACE2 (Figure 3A,C). Furthermore, the presence of VHH-72 stabilized the ACE2 receptor, though residues 103–105 and 133–140 displayed elevated flexibility (Figure 1 and Figure 3), suggesting localized conformational dynamics despite the global stabilizing effect.

**Figure 4:**
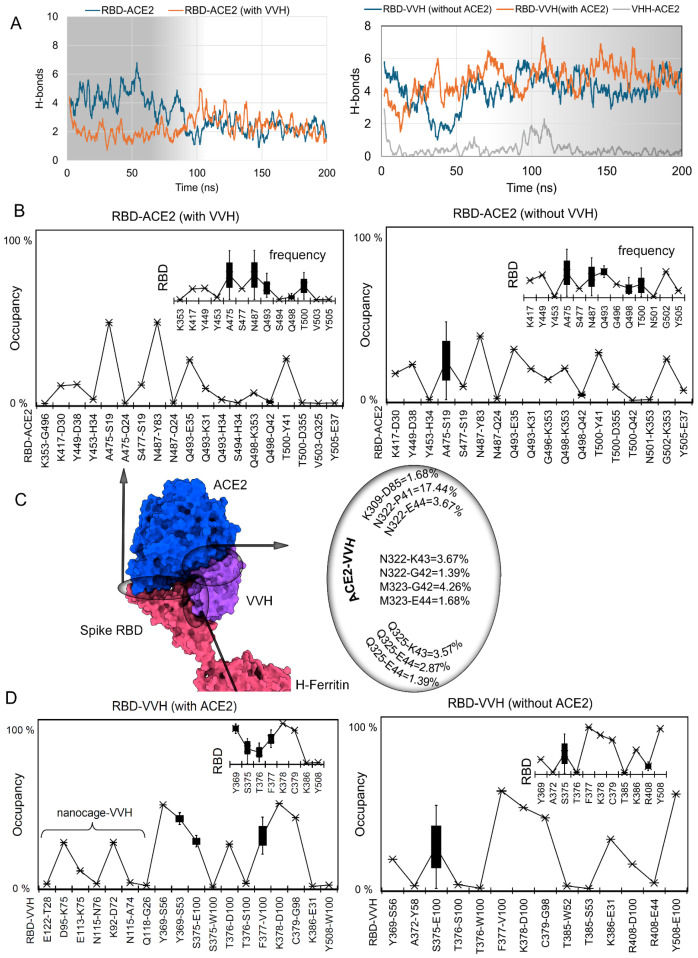
Frequency of interaction of VVH-72 with the SARS-CoV-2 Spike RBD over the ferritin nanocage. (**A**) Protein–protein or –antibody interactions (PPIs, H-bonds) from VVH-72-ACE2 and VVH-72 (apo-form) with the Spike RBD during the 200 ns. In addition, the interactions of ACE2-VVH-72 over the ferritin nanocage were monitored. (**B**) Influence of VVH-72 over the high-occupancy interaction > 1% (from 200 ns of MDS) for RBD-ACE2. The recurring RBD residues interacting with different amino acids of ACE2 were evaluated in terms of their frequency. (**C**) Structural representation of the Spike RBD-antibody-ACE2 complex over the ferritin nanocage. The right panel represents interacting residues for ACE2-VVH-72. ACE2 is shown in blue, VVH-72 in purple, and the S RBD in red. (**D**) The high-occupancy interaction > 1% of the frequency (from 200 ns) for RBD-VVH-72 in the presence or absence of the ACE2 receptor. The recurring RBD residues interacting with different amino acids of VVH-72 were evaluated in terms of their frequency. Occupancy defines a hydrogen bond interaction lasting more than 1% of the 200 ns MD simulation, computed using a donor–acceptor distance cutoff of 3.5 Å and donor–hydrogen–acceptor angle cutoff of 160–180°.

Measuring PPIs between ACE2 and RBD revealed that the presence of VVH-72 in the system induced the receptor to form a stable interaction with the RBD, compared to the system without an antibody (Figure 4A and Video S4). After comparing the protein–antibody interactions (H-bonds) over VVH-72-ACE2 and VVH-72 (apo-form) with the Spike RBD, it can be postulated that the presence of a receptor induces binding, though with different conformational switches resulting in fluctuations (Figure 4A). Toward 100 ns of MD simulation time, the ACE2-VVH-72 binding partnership over the ferritin nanocage formed many interactions that could enhance ACE2-RBD pairing (Figure 4A). Furthermore, to identify the difference in VVH-72 or ACE2 residues interacting with the RBD in the presence or absence of the respective partner, we computed individual residue binding occupancies (Figure 4).

Intermolecular interacting residues of the RBD-ACE2 complex in the presence or absence of monoclonal antibodies revealed that Spike RBD amino acids A475, N487, Q493, Q498, and T500 commonly interacted with the ACE2 receptor (Figure 4B). The following RBD-ACE2 residue pairs in the absence of the VVH-72 antibody, A475-S19, N487-Y83, Q493-E35, T500-Y41, G502-K353, Y449-D38, Q498-K353, Q493-K31, K417-D30, and G496-K353, were found to produce high-occupancy interactions (>10% occupancy). Though fewer interactions for RBD-ACE2 were observed in systems with VVH-72, most of the interacting pairs were found to be common in the apo-system, except for Q493-E35 and S477-S19 (Figure 4B and Video S4). High-frequency RBD residues such as Y369, S375, T376, F377, K378, C379, K386, and Y508 were found commonly interacting with VVH-72 in the presence or absence of ACE2 in the system (Figure 4D). In addition, the A372, T385, and R408 residues were found interacting with VVH-72 in the system with ACE2, and a few region of the monoclonal antibody were found interacting with different monomers of the H-ferritin nanocage.

The SARS-CoV-2 Spike RBD over the H-ferritin nanocage allowed us to identify different sites to dock with ACE2 or VVH-72 (Figure 4). When comparing these interacting residues with the X-ray structure (pdb id.: 6waq [41]), VVH-72 residues such as S56, G98, E100, W100, and V100, which emerged in docking with the S RBD, were found to be in common (Figure 4D). The RBD mutants S369Y and T372A from different SARS-CoV-2 strains were shown to have common interactions with VVH-72 in our findings and the X-ray structure, along with further common interactions (K378, F377, C379, Y508, and T372). In addition, S375 forming a high-occupancy interaction with a monoclonal antibody when mutated (S375F), reducing the Spike RBD-VVH-72 affinity [35,36,37].

### 2.3. Adopted Spike RBD Network with Receptor or Antibodies When Displayed over Nanocage

**Figure 5:**
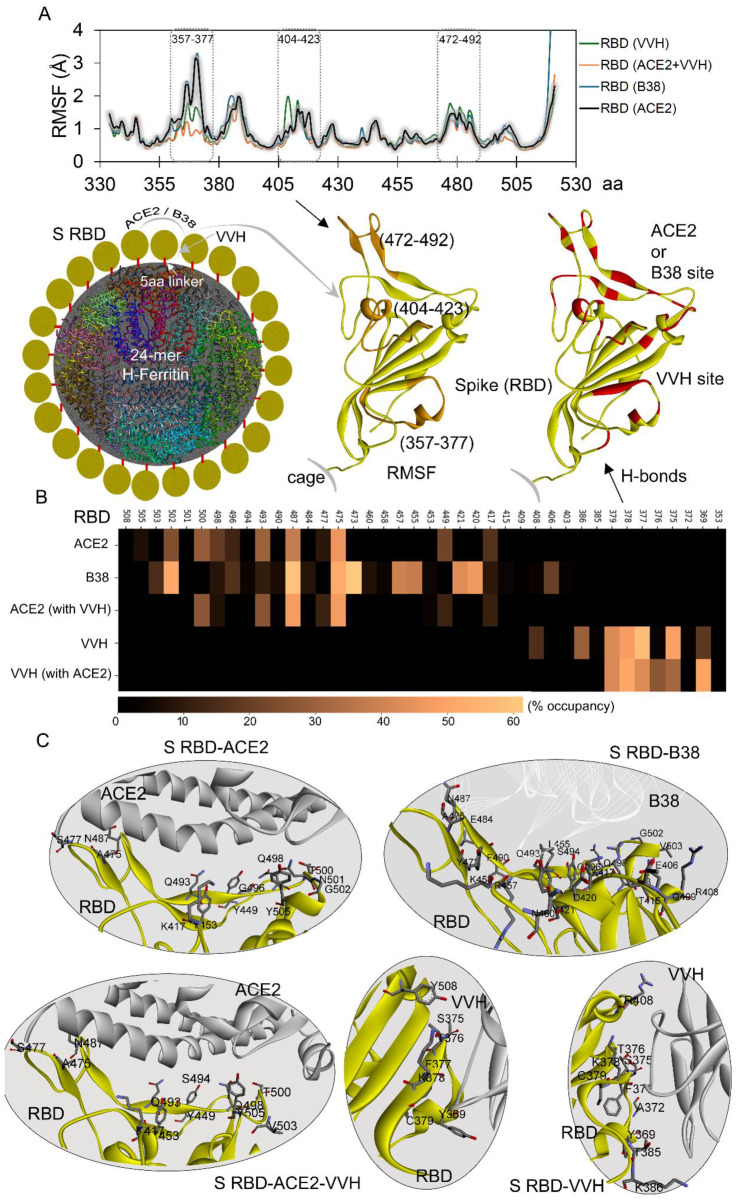
The SARS-CoV-2 Spike RBD interaction pattern and dynamics change with the receptor and antibodies upon display over the nanocage. (**A**) RMSF analysis of the Spike RBD in complex with ACE2, VVH-72, or B38 revealed distinct dynamic fluctuations. Key regions (357–377, 404–423, and 472–492 residues) exhibiting significant flexibility variations are highlighted in orange over the RBD structure (top panel). The lower panel illustrates 24 H-ferritin monomers (represented in different colors) displaying S RBD (in yellow), highlighting binding sites (in red) for ACE2, B38, and VVH-72, along with residue ranges showing dynamic differences. (**B**) Heatmap depicts high-occupancy interactions (more than 1% over 200 ns MD simulations) between RBD and receptors/antibodies (computed using matplotlib [43,44]). The upper panel shows RBD structures with labeled residues involved in hydrogen bonding with ACE2 or antibodies. (**C**) Structural models (retrieved from end of MDS) demonstrating high-occupancy interactions between the Spike RBD and its binding partners (ACE2, B38, or VVH-72) identified over 200 ns of MD simulation time, emphasizing key interfacial residues (represented as stick). VVH-72 or ACE2 is shown in gray, B38 in silver, and S RBD in yellow.

Despite sharing a common binding interface with the ACE2 receptor and the B38 antibody, the Spike (S) RBD exhibited distinct, dynamic behavior. In addition, a similar structural dynamic was observed for the VVH-72 antibody in the presence or absence of ACE2. Residue-level flexibility analysis (RMSF based on Cα atoms) revealed significant differences in certain S RBD regions upon binding to ACE2, B38, or VVH-72 (Figure 5A). Notably, residues 357–377, 404–423, and 472–492 displayed marked flexibility variation, along with the C-terminal region. When comparing these S RBD residues’ flexibility from our simulated models with receptors or antibodies with the apo-systems [37], it was observed that the presence of ACE2 or B38 stabilized the 472–492 residues, and the VVH-72 antibody showed significant stability in the 357–377 region (Figure 5A). Reduced fluctuation in these RBD residues correlated with specific interaction sites on the S domain when bound to either the receptor or antibodies (Figure 5B). To further elucidate binding differences and identify hotspot protein–protein interactions, we generated a heatmap [43,44] of high-occupancy networks (>1% occupancy over 200 ns MD simulations; Figure 5B).

Most high-affinity S RBD residues interacting with ACE2 were found to be engaged with B38, though a reverse behavior was lacking. Key residues A475 and N487 formed high-occupancy interactions with both ACE2 and B38, while D420, Y473, and G502 were specific to B38 (Figure 5B). For the systems with the VVH-72 antibody, residues such as F377, K378, and C379 participated in interaction regardless of ACE2’s presence. Specifically, Y369 mediated docking with VVH-72 in the presence of ACE2, whereas S375 was involved in its absence (Figure 5B). Analysis of the binding interfaces of the B38 and VHH-72 antibodies revealed distinct interaction patterns with the Spike RBD (Figure 5C). While the B38 mAb occupied the entire RBD interface responsible for ACE2 docking, effectively blocking receptor binding, the VHH-72 antibody showed no significant interference with RBD-ACE2 interactions (Figure 5C). Similarly, ACE2 binding lacked any substantial influence over S RBD’s interaction with VHH-72, further highlighting the differential binding mechanisms of these antibodies (Figure 5C).

Our MD simulations identified key O-glycosylated residues (O-glycosites) in the Spike RBD that participate in hydrogen bonding interactions with both antibodies and the ACE2 receptor. Notably, residues T376 and S477 identified as O-glycosites in experimental studies formed high-occupancy hydrogen bonds, suggesting potential roles in binding even in non-glycosylated simulation (Figure 5). These T376 and S477 amino acids emerged as critical glycosites in a human cell-expressed RBD, while T415 and S447 were predominant in an insect cell-expressed RBD (Figure 5). Protein glycosylation is the most common process of post-translational modification (PTM) in viruses [11,45,46]. Glycosylation promotes viral protein folding and subsequent trafficking as well as modulates their interactions with the receptors, which is followed by innate and adaptive immune response affecting the host recognition, viral replication, and infectivity [45,46,47,48]. According to the cryo-electron microscopy and topology data, the SARS-CoV-2 genome encodes a large number of highly glycosylated proteins, and their glycosylation extensively impacts the host recognition, penetration, binding, recycling, and pathogenesis [10,46]. Based on the in silico topology, the exact glycosites have been reported for Spike (S), envelope (E), membrane (M), and ORF3a proteins to date [10,46]. Since the S protein on the surface of the virus can recognize and bind to the ACE2 receptor, it determines the infectivity and transmissibility of SARS-CoV-2 [46,49]. A study by Grant et al. [49,50] indicates that the glycans shield approximately 40% of the protein surface of the S glycoprotein trimer from antibody recognition. While the degree of shielding of the underlying protein surface is relatively insensitive to the glycan type, it can certainly affect the innate immune response by altering the ability of collectins and other lectins of the immune system to bind the S glycoprotein efficiently and neutralize the virus, and it may overall impact the adaptive immune response by altering the number of viable HLA antigens [50,51].

## 3. Materials and Methods

H-ferritin constructs connected by various-sized linkers with the Spike RBD were generated in our previous study [37]. To achieve the proof-of-concept objectives, the coordinates of the Spike RBD-5aa S-Linker (GGGGS)-ferritin nanocage sampled after 100 ns of MD simulation [35,37] were chosen as a starting material. The 3D structure of the H-ferritin represented by pdb id.: 2fha [52] was chosen for the generation of 24 monomers containing nanocages, and the molecular models were prepared using the Proteins, Interfaces, Structures and Assemblies (PDBePISA) server [35,53]. The active form of the RBD (‘up’ conformation) structure (pdb id. 6lzg [36,37]) with a T333-P521 residue range was considered for docking with the ACE2 receptor or monoclonal antibodies. The optimal 5-amino-acid peptide (GGGGS) was used as the linker to stabilize the orientation of the Spike RBDs in the ‘up’ active conformation over the H-ferritin nanocages. To evaluate the ACE2 or monoclonal antibodies (B38 or VHH-72) with a Spike RBD-ferritin complex [37], the structures were superimposed onto their individual equivalent crystallization-state conformations from the PDB structures (pdb ids.: 6lzg [36,37], 7bz5 [40], and 6waq [41]). The optimized Spike RBD-ferritin structure after 100 ns [37] was used as a scaffold or template for superimposition, along with maintaining the mAbs conformation. Subsequently, the individual structures sampled from previous MD simulations [37] were also superimposed with their native structures using the Molecular Operating Environment (MOE; Chemical Computing Group Inc., Montreal, QC, Canada) package [54], maintaining the coordinates of the host receptor or antibodies. For structural superposition, a weighted non-linear optimization approach within the MOE (Chemical Computing Group Inc., Montreal, QC, Canada) package was employed to compute the optimal rigid-body transformations that maximize the alignment of protein atomic coordinates. This optimization minimizes the Mean Square Distance (MSD) between equivalent atoms in the compared structures.

The equilibrated models of the nanocage–linker–Spike (RBD) complexes were produced with the energy minimization protocol of the MOE (Chemical Computing Group Inc., Montreal, QC, Canada) package with default settings, using the CHARMM27 forcefield [55]. The resulting systems were then subjected to MD simulations using the GROMACS 4.6.5 [56] package. The parameters were generated using the CHARMM27 forcefield [55], each nanocage-linker-Spike (RBD) complex was centered in a dodecahedron simulation box, and the system was solvated using the simple point charge (spc) water model. The protein was placed in the box at least 10 Å from the box edge, periodic boundary conditions were formed in all directions, and the Na^+^ and Cl^−^ counter ions were added (corresponding to a physiological salt concentration of 150 mM) to the system for charge equilibration. The structure was first subjected to 50,000 steps of steepest-descent energy minimization in order to minimize the total potential energy and obtain the local minimum.

The particle mesh Ewald (PME) method [57] using a threshold of 1 nm was employed to account for the long-range electrostatic interactions, and the LINCS algorithm [58] was used to restrain the bond length. A threshold of 10 Å was used for the Coulomb electrostatic and van der Waals interactions. The thermodynamics ensemble nPT (isothermal isobaric ensemble) was applied to equilibrate each system for 1000 ps. Temperature and pressure were kept constant at 300 K and 1 bar by a velocity-rescaling thermostat [59] and the Parrinello–Rahman barostat [60], respectively. A leapfrog integrator [61] was used to integrate the equations of motion, while a time interval of 10 ps was used to save the trajectories for further analysis. MD simulations were carried out for 200 ns where systems reached the stabilized state, and trajectories were analyzed using GROMACS and visual molecular dynamics (VMD) tools [62]. The hydrogen bond interactions were defined using the donor–acceptor distance cutoff of 3.5 Å and the donor–hydrogen–acceptor angle cutoff of 160–180°. For visualization of protein structures and to analyze the molecular dynamics trajectories, the MOE (Chemical Computing Group Inc., Montreal, QC, Canada), BIOVIA Discovery Studio (DassaultSystèmes, BIOVIA Corp., San Diego, CA, USA), and VMD tools [62] and ChimeraX [63] were utilized. In addition, heatmap plots demonstrating protein–protein or protein–antibody interactions were generated using the matplotlib [43] library and Jupyter [44] packages.

## 4. Conclusions

Thermostable and biocompatible ferritin nanocages forming spherical self-assemblies have shown dual mechanisms, such as transporting molecules making potential usage of their hollow cavity, and multivalent antigen display over their surface, which can induce a cellular response. When implementing the MD simulation technique, we traced adopted structural dynamics within the surface SARS-CoV-2 Spike RBD with ACE2 and monoclonal antibodies (B38 and VVH-72) when presented over the H-ferritin nanocage. Among the studied mAbs, the conventional B38 antibody binds directly to the surface of the Spike RBD, whereas VVH-72 does not interfere with Spike-ACE2 binding. During MD simulation, the RBD that interacts with the ACE2 receptor maintains the ‘up’ active state, and the presence of VVH-72 mAb in the system maintains such a conformation. The interaction map between RBD-ACE2 and -B38 suggests that the nanocage constructs with antibodies formed almost a double affinity with the S RBD. Specifically, the VH chain from the B38 antibody contributed heavily to interactions with the RBD over the ferritin nanocage. Despite binding site overlap between ACE2 and B38, the RBD exhibited partner-specific conformational dynamics. The Cα-based RMSF analysis identified distinct flexibility patterns in key RBD subdomains when complexed with ACE2 versus neutralizing antibodies (B38 or VVH-72), suggesting specific allosteric effects. In addition, for both B38 (VH and VL) chains, an asymmetric fluctuation was observed, suggesting differential flexibility in their interaction with the RBD. Our findings demonstrated that the studied systems achieve stable states, though the structural interpretations could benefit from additional MD replicates to ensure robustness.

Identifying the PPIs or protein–mAbs interactions revealed that simulated systems have a strong correlation with validated structures, suggesting that modeled ferritin constructs can potentially mimic natural systems. In particular, the high-frequency networking RBD residues defining the pharmacophore when displayed over nanocage molecules were identified: Y473, N487, A475, D420, Y421, R457, L455, R457, G502, E406, G496, V503, and Q493 evolved from the heavy and light chains of B38, respectively. The Y369, S375, T376, F377, K378, C379, K386, and Y508 RBD residues were found commonly interacting with VVH-72 regardless of whether there was ACE2 in the system. In addition, the A475, N487, Q493, Q498, and T500 RBD residues were common in interacting with ACE2 in the presence or absence of the mAbs. Our findings reveal that ferritin nanocages functionalized with the SARS-CoV-2 Spike RBD exhibit dynamic structural states, potentiating enhanced interactions with host-receptor and neutralizing antibodies. Overall, protein–protein/mAbs hydrogen bonds can guide the understanding of interfaces of different components, which can be further evaluated using free energy calculations, such as MM/PBSA (Molecular Mechanics Poisson–Boltzmann Surface Area). This study analyzed the binding patterns of the Wuhan-Hu-1 strain with ACE2 and mAbs on nanocages, highlighting ferritin-based techniques as promising SARS-CoV-2 vaccine candidates, with future potential to explore variant RBD dynamics with receptors or antibodies. These computational data can be further validated through in vivo or in vitro experiments, such as co-immunoprecipitation (co-IP), surface plasmon resonance (SPR), etc. We believe that these versatile positions from the ferritin-RBD conjugates define a robust predicted platform for the development of pre-fusion-stabilized, broadly protective RBD-based vaccines.

## Data Availability

All data generated or analyzed in this study are included in the article and Supplementary files. Additional datasets generated for MD simulations are available from the corresponding authors upon reasonable request.

## Supplementary Materials

The following supporting information can be downloaded at https://www.mdpi.com/article/10.3390/ijms26157047/s1.
